# Absence of cytotoxic and inflammatory effects following in vitro exposure of chondrogenically-differentiated human mesenchymal stem cells to adenosine, lidocaine and Mg^2+^ solution

**DOI:** 10.1186/s40634-019-0185-5

**Published:** 2019-04-15

**Authors:** Andrew McCutchan, Geoffrey P. Dobson, Natalie Stewart, Hayley L. Letson, Andrea L. Grant, Ivana-Aleksandra Jovanovic, Kaushik Hazratwala, Matthew Wilkinson, Peter McEwen, Jodie Morris

**Affiliations:** 1 Department of Haematology and Bone Marrow Transplantation, Townsville Hospital, Townsville, Australia; 2 Heart, Trauma and Sepsis Research Laboratory, College of Medicine and Dentistry, James Cook University, Townsville, Australia; 3 The Orthopaedic Research Institute of Queensland, 7 Turner St, Pimlico, Townsville, Q 4812 Australia

**Keywords:** Chondro-MSC, Chondrocyte, ALM, Tranexamic acid, Viability, Inflammation

## Abstract

**Background:**

ALM solution, a combination of adenosine, lidocaine and Mg^2+^, is an emerging small volume therapy that has been shown to prevent and correct coagulopathy and surgery-related inflammation in preclinical models, though its application in orthopaedic surgery is yet to be demonstrated. The effect of ALM solution on chondrocytes is unknown. The aim of this preliminary study was to investigate the effect of ALM solution on viability and inflammatory responses of chondrogenically-differentiated human bone marrow-derived mesenchymal stem cells (chondro-MSC), in vitro*.*

**Methods:**

Chondro-MSC were exposed to media only, saline (0.9% NaCl or 1.3% NaCl) only, or saline containing ALM (1 mM adenosine, 3 mM lidocaine, 2.5 mM Mg^2+^) or tranexamic acid (TXA, 100 mg/ml) for 1 or 4 h. Responses to ALM solutions containing higher lidocaine concentrations were also compared. Chondrocyte viability was determined using WST-8 colorimetric assays and inflammatory cytokine (TNF-α, IL-1β, IL-8) and matrix metalloproteinases (MMP-3, MMP-12, MMP-13) concentrations using multiplex bead arrays.

**Results:**

The viability of chondro-MSC was significantly greater after 1 h treatment with ALM compared to saline (96.2 ± 7.9 versus 75.6 ± 7.3%). Extension of exposure times to 4 h had no significant adverse effect on cell viability after treatment with ALM (1 h, 85.4 ± 5.6 *v* 4 h, 74.0 ± 15.2%). Cytotoxicity was evident following exposure to solutions containing lidocaine concentrations greater than 30 mM. There were no significant differences in viability (80 ± 5.4 *v* 57.3 ± 16.2%) or secretion of IL-8 (60 ± 20 *v* 160 ± 50 pg/ml), MMP-3 (0.95 ± 0.6 *v* 3.4 ± 1.6 ng/ml), and MMP-13 (4.2 ± 2.4 *v* 9.2 ± 4.3 ng/ml) in chondro-MSC exposed to saline, ALM or TXA.

**Conclusions:**

Short-term, in vitro exposure to clinically-relevant concentrations of ALM solution had no adverse inflammatory or chondrotoxic effects on human chondro-MSC, with responses comparable to saline and TXA. These findings provide support for continued evaluation of ALM solution as a possible therapeutic to improve outcomes following orthopaedic procedures.

## Background

An ageing population and rise in prevalence of comorbid chronic disease is projected to drive a continued global increase in demand for elective arthroscopy and arthroplasty procedures (Inacio et al. 2017a, b, Holtedahl et al. 2018). As with any surgery, there are a number of potential complications following orthopaedic surgical procedures including local bleeding, coagulopathy, inflammation and tissue trauma (Cheuy et al. 2017, Friberger Pajalic et al. 2018). While inflammation and immune modulation are essential for tissue repair, sustained or overexpression of these responses can lead to further bleeding, joint damage, infection and arthrofibrosis (Eming et al. 2007, Dobson 2015, Järvinen and Guyatt 2016, Werthel et al. 2017).

The anti-fibrinolytic tranexamic acid (TXA) has become increasingly popular in orthopaedic procedures to improve visualization and reduce some of these complications (Karaaslan et al. 2015, Demos et al. 2017, Abdel et al. 2018). However, debate continues regarding the effect of TXA on articular cartilage and post-operative inflammation (Jang et al. 2014, Goyal et al. 2017, Fillingham et al. 2018, Grant et al. 2018, Moskal and Capps 2018, Parker et al. 2018). A combination of adenosine, lidocaine and Mg^2+^ (ALM solution) is an emerging small volume therapy that has been shown to prevent and correct coagulopathy and surgery-related inflammation, and reduce the trauma of surgery in several preclinical models (Dobson and Letson 2016). In contrast to TXA, which acts as a downstream anti-fibrinolytic (Reed and Woolley 2015, Dobson et al. 2018), ALM appears to correct coagulopathy upstream at the thrombomodulin complex located on the endothelium (Dobson et al. 2015, Dobson and Letson 2016). In addition, ALM has been shown to be a potent anti-inflammatory following hemorrhagic shock, traumatic brain injury, abdominal surgery and polymicrobial sepsis (Granfeldt et al. 2014, Griffin et al. 2014, Letson and Dobson 2015, Griffin et al. 2016, Davenport et al. 2017, Letson and Dobson 2017a, b Letson and Dobson 2018a, b). The application of ALM solution in orthopaedic surgical procedures is yet to be described. However, based on preclinical models of major surgery (Dobson and Letson 2016, Davenport et al. 2017), ALM solution has the potential to correct coagulopathy and reduce tissue-damaging inflammatory responses following joint injury and during orthopaedic procedures. As a precursor to evaluating ALM solution in orthopaedic models, preliminary in vitro studies are necessary to determine if clinically-relevant concentrations of ALM solution exert potentially adverse effects on human chondrocytes. Due to difficulties associated with isolating and maintaining primary human chondrocytes cultures at sufficient cell densities for in vitro characterisation assays, chondrogenically-differentiated human bone marrow-derived mesenchymal stem cells (chondro-MSC) were used as a substitute for these proof-of-concept studies. Our aim was to investigate the effect of ALM solution on viability and inflammatory responses of chondro-MSC, in vitro*.* We hypothesised that ALM solution would show no cytotoxic or stimulatory effect on chondro-MSC, and would be comparable to saline and TXA.

## Methods

### Approvals

Informed consent was obtained prospectively from all participants, and the study was approved by the Institutional Human Research Ethics Committee (HREC/15/QTHS/39). The research undertaken strictly adhered to the Code of Ethics (Declaration of Helsinki) of the World Medical Association for trials involving humans.

#### Cell isolation, culture conditions and in vitro chrondrogenic differentiation

Ten patients (8 male, 2 females; age range 20–66 years) undergoing a bone marrow biopsy procedure as part of a routine assessment of their remission status for a haematological condition were recruited to participate in the study following informed consent. The buffy coat from an aspirated bone marrow sample (1 ml) was isolated from each patient and washed in HBSS. Nucleated cells (2 × 10^7^) were plated in 75cm^2^ culture flasks (T75) with Expansion media (Dulbecco Modified Eagle Medium, DMEM + GlutaMAX, Thermo Fisher Scientific, Scoresby, VIC) supplemented with foetal bovine serum (10%, Sigma-Aldrich, Castle Hill, NSW), human fibroblast growth factor (2 ng/ml, Miltenyi Biotec, Macquarie Park, NSW), and penicillin/streptomycin (1%, Thermo Fisher Scientific), and cultured for 24 h at 37 °C in a humidified atmosphere of 5% CO_2_. Non-adherent cells were removed after 24 h culture, with further media changes performed three times a week (Mareschi et al. 2012). To promote an even distribution of MSC, at day 14 the cells were detached with TrypLE™ Express (Thermo Fisher Scientific), washed and transferred to new flasks, with secondary cultures reaching 80–90% confluence within 7 days of subculture. To minimise any loss of differentiation potential, MSC were not cultured beyond the second passage.

**Fig. 1:**
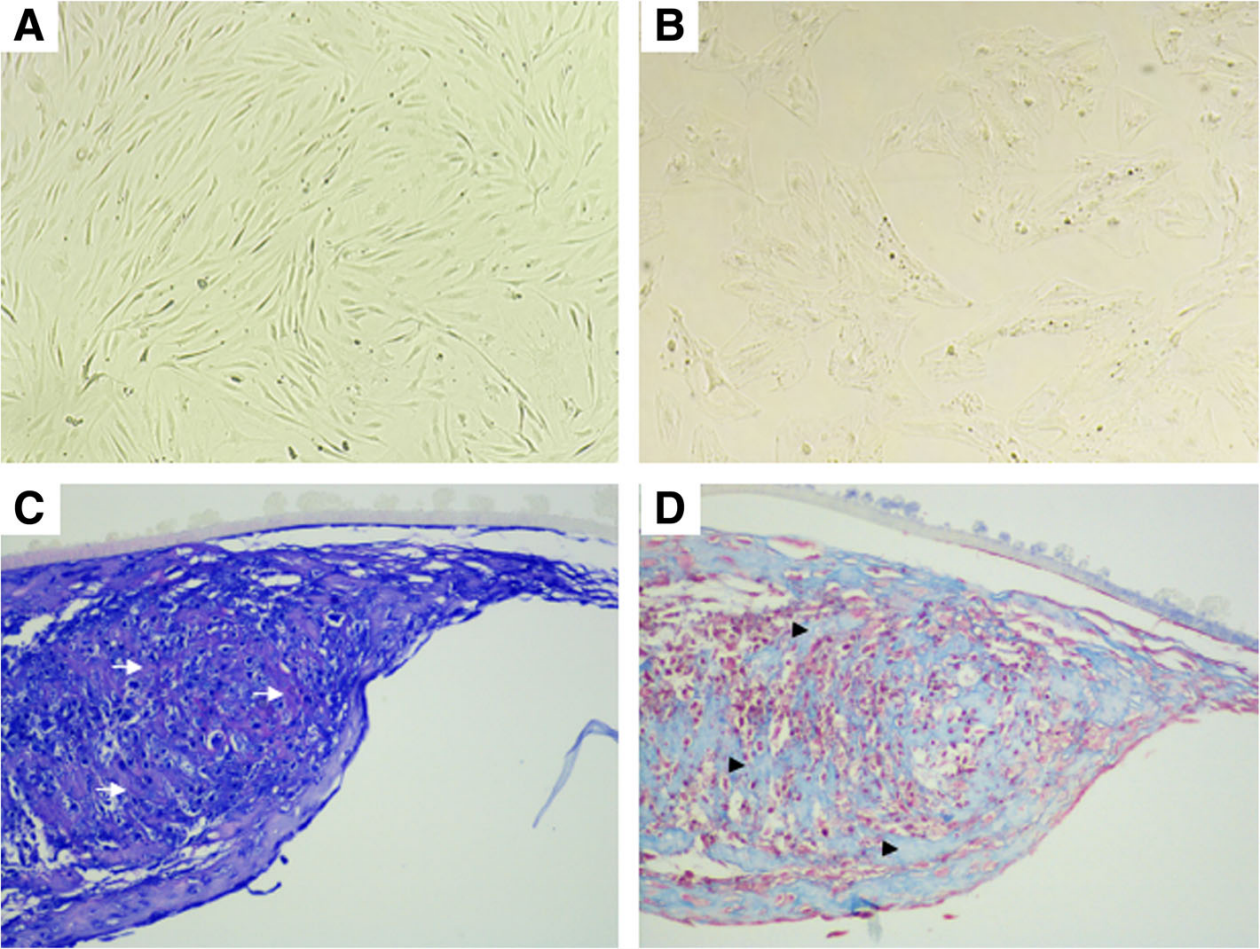
Chondrogenic differentiation of human MSC. Cellular morphology changes were evident in human MSC (**a)** prior to, and (**b**) following culture in chondrogenic differentiation media for 7 days, with the transformation of spindle-shaped MSC to rounded, polygonal chondrocytes (magnification, 10x). Chondrogenic differentiation was confirmed histologically, with chondrocyte aggregation and early structural organisation of a collagenous matrix evident. Representative formalin-fixed, paraffin-embedded sections of chondro-MSC were stained with (**c**) toluidine blue to demonstrate sulphated glycosaminoglycans deposition (white arrow) or (**d**) Picro-Mallory trichrome stain highlighting deposition of collagen fibres (black arrowhead) within the extracellular matrix (magnification, 20x)

In vitro chondrogenesis was performed according to published protocols (Solchaga et al. 2011). Expanded MSC were trypsinised, washed and 7.5 × 10^5^ cells transferred to new T75 flasks. Chondrogenic differentiation was induced by treatment with STEMPRO™ Chondrogenesis Differentiation medium (Thermo Fisher Scientific) (Kalamegam et al. 2016). Monolayers were incubated at 37 °C in 5% CO_2_ with differentiation media changes performed three times a week. After 7 days, the monolayer was observed microscopically to confirm chondrocyte morphology. Differentiation was regarded as > 90% conversion of a spindle-shaped, fibroblastic MSC morphology to a rounded, polygonal morphology consistent with chondrocyte monolayers (Fig. 1a, b). Chondro-MSC were not cultured beyond first passage to avoid de-differentiation. Following visual confirmation, chondro-MSC were trypsinised, washed and counted. Chondro-MSC were transferred to the wells of a 96-well plate at high cell densities (> 5 × 10^3^) to avoid de-differentiation and were subsequently used for assessment of viability and cytokine production in response to ALM and TXA exposure as described below.

#### Histology

To confirm chondrogenic differentiation of MSC using the culture conditions described above, 5 × 10^5^ MSC were placed in the wells of a 24-well Transwell® plate (Sigma-Aldrich) and differentiation media was added, with media changes performed three times a week (Murdoch et al. 2009). After 21 days, the Transwell membrane was removed and fixed in 4% paraformaldehyde, processed histologically and paraffin-embedded sections stained with hematoxylin and eosin (H&E), toluidine blue and Picro-Mallory trichrome. Chondrocyte aggregation and early stages of matrix structural organisation was in evident histologically. Sections stained with toluidine blue demonstrated the presence of negatively charged proteoglycans, whilst blue colouration in trichrome-stained sections confirmed the deposition of a collagenous matrix between chondrocytes (Fig. 1c, d).

#### Treatments

Adenosine (A9251), Lidocaine-HCl (L5647), and MgSO_4_ (M7506) were purchased from Sigma-Aldrich. Chondrocytes were incubated 1) in media only (no Tx), 2) saline (0.9% NaCl or 1.3% NaCl) only, 3) adenosine (1 mM) alone, lidocaine (3 mM) alone, or Mg^2+^ (2.5 mM) alone, and 4) ALM solution combined (adenosine 1 mM, lidocaine 3 mM, Mg^2+^ 2.5 mM). Given that 1% (~ 37 mM) and 2% (~ 74 mM) lidocaine solutions are known to be toxic to chondrocytes (Karpie and Chu 2007, Baker et al. 2011), we also examined ALM with 30 mM lidocaine (AL_30_M) and 60 mM lidocaine (AL_60_M) as internal controls. In addition, ALM solution (0.9% saline) was compared to TXA (100 mg/ml in 0.9% saline, Juno Pharmaceuticals, South Yarra, VIC). Since intra-articular doses of 1 to 3 g of TXA in 20 ml saline (50–150 mg/ml) are used in our institution and by others (Ishida et al. 2011, Tuttle et al. 2014), a concentration of 100 mg/ml TXA was selected for comparison to ALM solution in the current study. Chondro-MSC were exposed to treatments for 1 or 4 h (37 °C, 5% CO_2_) prior to processing for assessment of viability or secreted inflammatory cytokines, chemokines and matrix metalloproteinases (MMP). Exposure times of 1 and 4 h, were selected to reflect the mean surgical times associated with arthroplasty procedures (Weber et al. 2018). The longer exposure time reflects the 4 h ALM infusion times used in previous in vivo studies to confer protection in preclinical models of trauma, sepsis and surgery (Griffin et al. 2014, Griffin et al. 2016, Letson and Dobson, 2017a, b).

#### Viability assay

Chondrocyte viability was assessed using a WST-8 colorimetric assay, according to manufacturers’ protocol (Cell Count Kit-8, Sigma Aldrich). The WST-8 assay assesses mitochondrial ability to reduce WST-8 into water-soluble tetrazolium salt. Briefly, chondro-MSC were enzymatically lifted from flasks and dispensed into a 96-well plate at 5 × 10^3^ cells/well. After incubation for 24 h to allow attachment, ALM or individual components were added to appropriate wells for 1 or 4 h (37 °C, 5% CO_2_). After treatment, the medium was removed, fresh culture media added and plates incubated for a further 18 h (37 °C, 5% CO_2_). WST-8 (10 μl) was added and plates incubated for a further 4 h (37 °C, 5% CO_2_). Absorbance was measured at 450 nm, with a reference of 650 nm using a Multiskan EX microplate reader (Thermo Fisher Scientific). Cell viability was calculated using the ratio of the absorbance of treated (saline, ALM or individual components, TXA) cells to the absorbance of untreated (culture media only) cells, and data were expressed as percentages.

#### Cytokine, chemokine and MMP analysis

In a parallel set of experiments, the secretion of inflammatory cytokines, chemokines and MMP from chondro-MSC was compared in ALM- and TXA-treated cultures. Briefly, expanded MSC were enzymatically lifted from flasks and dispensed into a 96-well plate (5 × 10^4^ cells/well). After overnight incubation, expansion media was replaced with chondrogenic differentiation media, and cultures incubated for 7 days with differentiation media changes performed three times. Saline only (0.9%), ALM, or TXA solutions were added to appropriate wells for 1 h. Recombinant human IL-1β was purchased from Sigma Aldrich (I9401) and included in each experiment as a positive control (5 ng/ml; Forsyth et al. 2005). Chondro-MSC were washed, media replaced and cells incubated for a further 24 h prior to removal of cell-free supernatants from untreated and treated wells. Milliplex® Human Cytokine/Chemokine (HCYTOMAG-60 K, Lot #2929279) and MMP Magnetic Bead Panel 1 (HMMP1MAG-55 K, Lot #10101, Abacus ALS, Meadowbrook, Queensland) in combination with the Magpix® analyser (Luminex Corporation, Austin, Texas, USA) were used to measure levels of interleukin (IL)-8, IL-1β, tumor necrosis factor alpha (TNF-α), MMP-3, MMP-12 and MMP-13 in chondro-MSC culture supernatants. Assays were carried out according to manufacturer’s instructions with samples measured in duplicate.

#### Statistics

GraphPad Prism 7 was used for all data analysis. Data normality was assessed using Shapiro-Wilks test, with Levene’s test used to determine equality of variances. Viability between groups were compared using t-tests, univariant variance analysis (ANOVA, Tukey post-hoc analysis) or two-way ANOVA (Tukey post-hoc analysis) where appropriate. MILLIPLEX Analyst 5.1 software (Luminex Corporation, Austin, Texas, USA), which analyses data with a 5-parametric logistic weighted curve fit, was used to determine cytokine and MMP concentrations. Cytokine and MMP concentrations between groups were compared using Kruskal-Wallis test with Dunn’s post-hoc analysis. All values are expressed as mean ± standard error of the mean (SEM) with significance set at *P* < 0.05.

## Results

### ALM improves chondrocyte viability

**Fig. 2:**
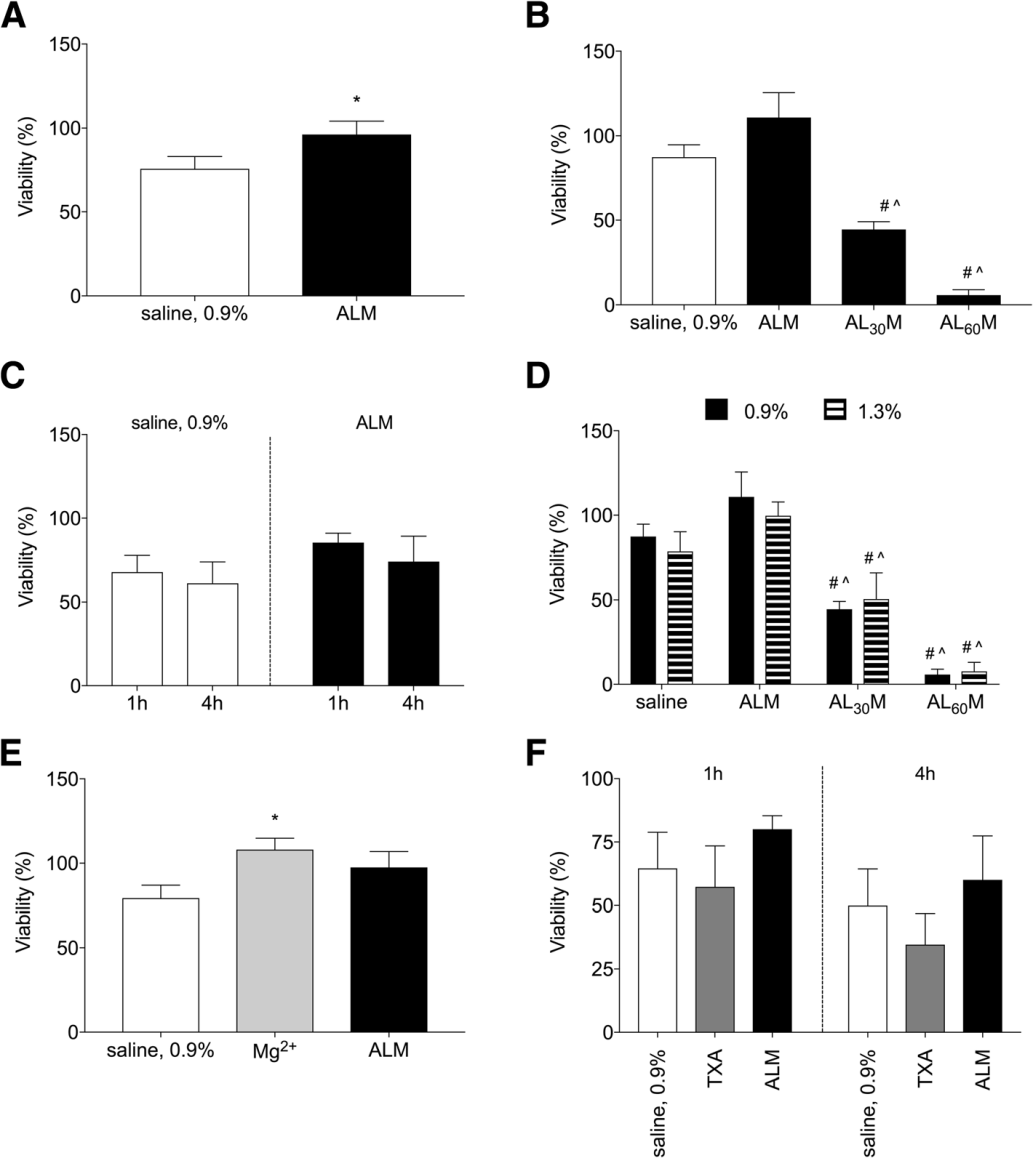
Effect of saline, ALM and TXA solutions on chondro-MSC viability, where cell viability is defined as the mean absorbance of treated (saline, ALM, TXA) cells normalised to the mean absorbance of untreated (culture media only) cells. **a** Compared to 0.9% saline, viability was significantly greater in cultures exposed to ALM solution for 1 h (*P* = .045, *n* = 13). **b** Increasing lidocaine concentrations within the ALM preparation to 30 mM (AL_30_M) and 60 mM (AL_60_M) resulted in significantly reduced viability (*P* < .001, *n* = 6, # compared to saline; ^ compared to ALM). **c** There were no significant differences in chondrocyte viability at 1 and 4 h exposure of chondrocytes to ALM solution. **d** No significant differences were observed between 0.9% and 1.3% saline (n = 6). **e** Compared to normal saline (0.9%), addition of Mg^2+^ significantly improved cell viability (*P* = .043, *n* = 11). **f** No significant differences were observed in the viability of cultures exposed to ALM solution or TXA for 1 (*P* = .47) or 4 h (*P* = .49; *n* = 5). Data is expressed as mean ± S.E.M. ALM, adenosine, lidocaine, and magnesium; MgSO_4_, magnesium sulphate; MSC, mesenchymal stem cells; TXA, tranexamic acid

The viability of chondro-MSC treated with ALM solution for 1 h was significantly greater than cultures exposed to normal saline (96.2 ± 7.9% *v* 75.6 ± 7.3%, *P* = .045, Fig. 2a). In contrast, chondrotoxicity was demonstrated by significantly reduced viability in cultures exposed to ALM solution where lidocaine concentrations were increased to 30 mM (44.5 ± 4.6%) or 60 mM (5.8 ± 3.1%, *P* < 0.001, Fig. 2b). There was no difference in the viability of ALM-treated chondro-MSC between 1 and 4 h of exposure (85.4 ± 5.6% *v* 74 ± 15.2%. *P* = .214, Fig. 2c).

Exposure of chondro-MSC to adenosine alone (1 mM) or lidocaine alone (3 mM) resulted in viability that was comparable to cultures exposed to saline vehicle (saline, 86.9 ± 4.9%; adenosine, 87.6 ± 5.6%; lidocaine 94.5 ± 7.1%; *P* = .93). In separate experiments, viability was significantly higher in chondro-MSC cultures exposed to normal saline with Mg^2+^ compared to saline alone (108 ± 6.8% *v* 79.3 ± 7.6%, *P* = .043, Fig. 2e). Viability was comparable between Mg^2^-supplemented saline and ALM-treated chondro-MSC cultures (108 ± 6.8% *v* 97.6 ± 9.4%, Fig. 2e).

Chondroprotection has previously been demonstrated in response to increased osmolarity in bovine cartilage explant cultures (480 mOsm, ~ 1.3% saline) (Amin et al. 2010). To investigate whether chondrocyte viability was affected by saline solution osmolarity in the current study, chondro-MSC were cultured in the presence of 0.9% or 1.3% saline only, ALM in 0.9% saline or ALM in 1.3% saline. No significant differences were observed in the viability of chondro-MSC exposed to either 0.9% or 1.3% saline (87.4 ± 7.3% *v* 78.6 ± 11.7%, *P* = .894). Similarly, the viability of chondro-MSC exposed to ALM in 0.9% saline was comparable to cultures exposed to ALM in 1.3% saline (*P* = .667, Fig. 2d).

### Effects of ALM and TXA on chondrocyte viability and inflammatory response

No significant differences were observed in the viability of chondro-MSC exposed to ALM or TXA for 1 h (80 ± 5.4% *v* 57.3 ± 16.2%, *P* = .47) or 4 h (60.1 ± 17.3% *v* 34.6 ± 12.3%, *P* = .49, Fig. 2f).

**Fig. 3:**
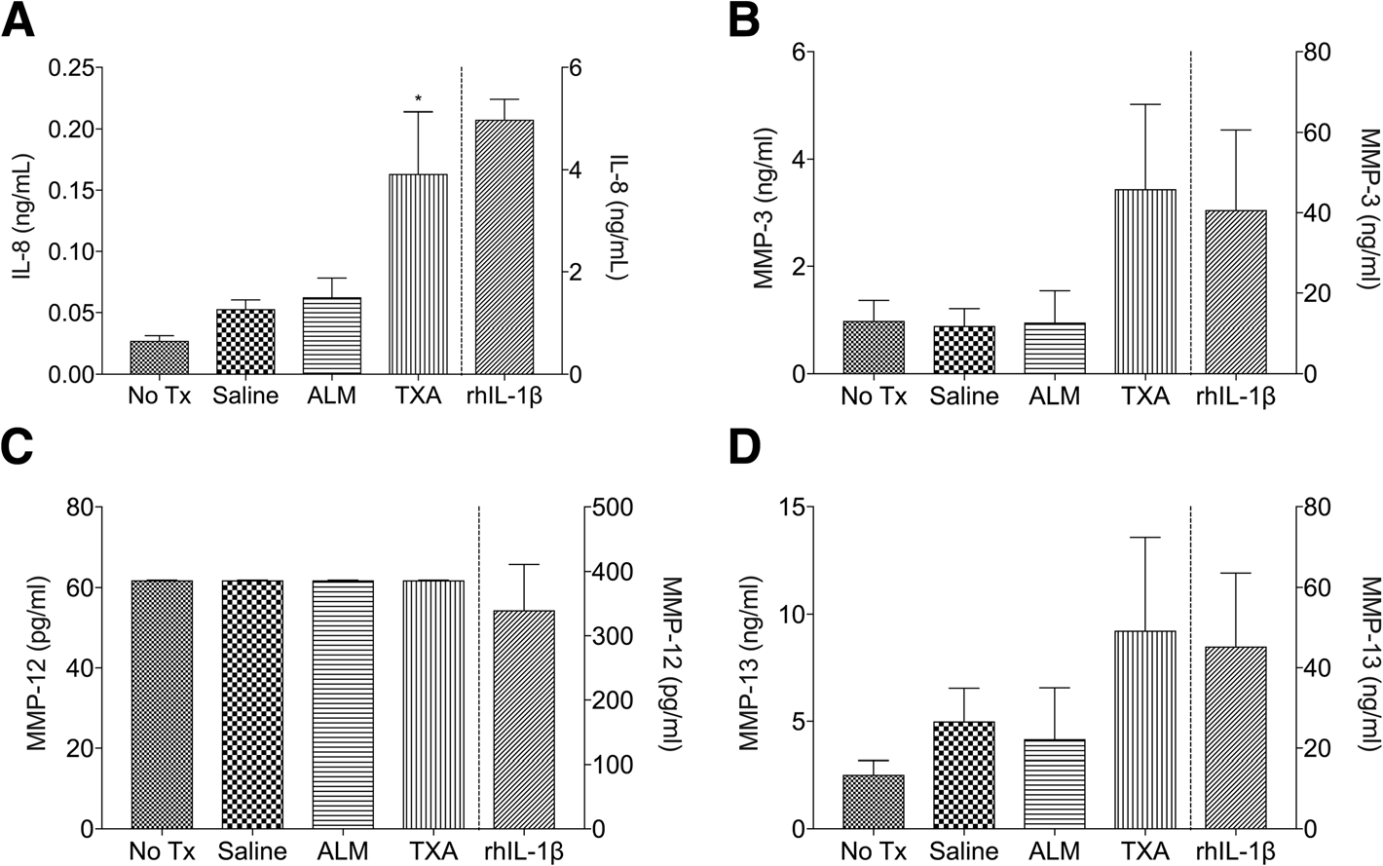
Concentration of (**a**) IL-8, (**b)** MMP-3, (**c**) MMP-12 and (**d**) MMP-13 in chondro-MSC cultures left untreated (culture media only) or exposed to 0.9% saline, ALM, TXA or recombinant human IL-1β. Data is expressed as mean ± S.E.M. *P < 0.05 compared to no treatment. ALM, adenosine, lidocaine, and magnesium; IL, interleukin; MMP, matrix metalloproteinase; MSC, mesenchymal stem cells; TXA, tranexamic acid

Cell-free culture supernatants were assessed for inflammatory cytokine and chemokines and MMP concentrations following exposure to ALM or TXA for 1 h (Fig. 3). Levels of IL-1β, TNF-α and MMP-12 were below the limit of detection for each treatment group, despite measurable levels in rhIL-1β-treated chondro-MSC cultures (IL-1β 3.0 ± 0.2 pg/ml, TNF-α 2.8 ± 0.5 pg/ml, MMP-12, 340 ± 71.2 pg/ml). In contrast, secretion of IL-8 was significantly increased in chondro-MSC cultures exposed to TXA (*P* = .044) compared to untreated (culture media only) cells (Fig. 3a). Levels of MMP-3 and MMP-13 were comparable for between treatment groups, however greater variability was observed in the concentrations of these MMPs in cultures exposed to TXA (Fig. 3b).

## Discussion

We report the absence of cytotoxic and inflammatory responses following short-term, in vitro exposure of human chondro-MSC to ALM solution. ALM solution is an emerging small-volume therapy containing a combination of adenosine, lidocaine and magnesium (Dobson and Letson 2016). At high concentrations, ALM solution arrests the heart and is currently in clinical use as a method for polarizing cardioplegia (Djabir et al. 2013). At 10-fold lower ‘non-arrest’ concentrations, ALM solution has been shown to correct coagulopathy, improve cardiac function, and reduce immune-inflammatory activation in response to sterile surgery and trauma in preclinical models (Granfeldt et al. 2014, Dobson et al. 2015, Letson and Dobson 2015, Davenport et al. 2017, Letson and Dobson 2017a, b, Letson and Dobson 2018a, b).

### ALM appears to be safe at low lidocaine concentrations

The present study found that ALM solution (3 mM or 0.08% lidocaine) significantly improved chondrocyte viability by 30% compared to normal saline. No benefit was found using a higher 1.3% hypertonic salt solution, which some groups have reported is more chondroprotective than normal saline (Amin et al. 2010). Consistent with the findings of previous studies using human chondrocytes (Karpie and Chu 2007, Baker et al. 2011), lidocaine at higher concentrations (30 and 60 mM) was toxic to chondro-MSC in the current study, with cell viability decreasing by 65% and 95%, respectively. The mechanisms for lidocaine toxicity at 1% (37 mM) or 2% (74 mM) concentrations, and other local anesthetics (e.g. bupivacaine, ropivacaine, mepivacaine), appear to be associated with impairment of chondrocyte metabolism, which leads to cell death (Kreuz et al. 2018). Dragoo and colleagues (Dragoo et al. 2012) also showed that 1% lidocaine in human monolayer chondrocyte cultures was associated a reduction of extracellular matrix molecules, glycosaminoglycan, and collagen content compared to controls after 3 h. Interestingly, Baker et al. (2011) showed that lidocaine toxicity could be attenuated with the addition of magnesium. In the present study, we found the addition of magnesium sulphate to normal saline led to 10% higher cell viability. The significance of using ALM solution compared to Mg^2+^ alone will be discussed in Potential Clinical Relevance section (see below).

### Chondrocyte viability comparable for ALM and TXA solution

Using a clinically relevant, intra-articular dose of TXA (100 mg/ml; Ishida et al. 2011, Tuttle et al. 2014), our study found no significant difference between normal saline, ALM and TXA after 1 h exposure, although mean chondro-MSC viability in TXA-exposed cultures was reduced by 15% and 29% compared to normal saline and ALM solution, respectively. Parker et al. (2018) recently demonstrated significant dose-response chondrotoxicity of TXA toward human chondrocytes using lower concentrations of TXA (40 mg/ml), with a 58% loss of viability in monolayers, and approximately 30% decrease in hydrogel-encapsulated 3D human chondrocyte cultures following 3 h exposure. Parker et al. (2018) and more recently, Ambra et al. (2017) recommended that intraarticular administration of TXA in orthopaedic surgery may be safe at TXA concentrations below 20 mg/ml (Parker et al. 2018). Notwithstanding these studies, standardised protocols for intra-articular use of TXA are lacking and debate continues regarding potential detrimental effects following its use in arthroscopy and other orthopaedic procedures where native articular cartilage remains (Jang et al. 2014, Goyal et al. 2017, Fillingham et al. 2018, Grant et al. 2018, Moskal and Capps 2018, Parker et al. 2018). Further in vivo studies are required to evaluate potential underlying dose-dependent mechanisms of TXA chondrotoxicity.

### Absence of inflammatory activation of chondrocytes exposed to ALM

Another interesting finding in our study was that ALM solution did not activate secretion of inflammatory mediators known to be involved in cartilage breakdown and joint deterioration (Chen et al. 2014, Pap and Korb-Pap 2015, Takahashi et al. 2015, Minguzzi et al. 2018) from human chondro-MSC cultures, with responses comparable to those exposed to normal saline (Fig. 3). No statistically significant difference was observed in inflammatory mediator concentrations between cultures exposed to either saline, ALM or TXA, although levels of IL-8 were significantly higher in chondro-MSC cultures treated with TXA compared to untreated cultures. IL-8 is expressed at increased levels in human osteoarthritic chondrocytes (Takahashi et al. 2015, Minguzzi et al. 2018) and is associated with: 1) production of IL-1β, IL-6, and TNF-α, 2) the induction of chondrocyte hypertrophy and differentiation, 2) the release of MMP-13, and 3) neutrophil-mediated inflammation and cartilage breakdown from generation of reactive oxygen metabolites (Lotz et al. 1992, Takahashi et al. 2015). It is noteworthy that TXA administration in patients undergoing TKA was recently demonstrated to lead to significant increases in plasma inflammatory cytokines during and after surgery (Grant et al. 2018).

### Potential clinical relevance

During orthopaedic surgical procedures, surgery-induced damage within joint tissues can activate chondrocytes in articular cartilage (Schulze-Tanzil 2009, Dobson 2015). Chondrocyte activation leads to increased production of pro-inflammatory cytokines such as IL-1β and TNF-α which, in the absence of homeostatic regulation, drive an inflammatory cascade that promotes upregulation of cartilage-degrading proteinases and chondrocyte apoptosis (Schulze-Tanzil 2009, Pap and Korb-Pap 2015, Minguzzi et al. 2018). Strategies to reduce secondary injury progression are actively being sought to improve regulation of the postoperative healing process and patient outcomes. Small-volume bolus and infusion of ALM solution has been shown to improve survival and prevent secondary injury progression in a number of different trauma states in small and large animal models of hemorrhagic shock (Granfeldt et al. 2014, Dobson and Letson 2016, Letson and Dobson 2017a, b), cardiac arrest (Djabir et al. 2013), polymicrobial sepsis (Griffin et al. 2014, Griffin et al. 2016), abdominal surgery (Davenport et al. 2017), and more recently traumatic brain injury (Letson and Dobson 2018a, b). The addition of magnesium to normal saline was found to increase chondro-MSC viability in the current study and is consistent with in vitro chondroprotective properties described by others (Baker et al. 2011). The analgesic properties of magnesium may be due to its ability to block NMDA receptors and potentially through inhibitory effects on calcium and potassium channels, thus augmenting neural transmission (Srebro et al. 2016). Magnesium has been evaluated as an alternative intra-articular analgesic in arthroscopic knee surgery conferring better pain relief than placebo (Bondok and Abd El-Hady 2006), and showing a synergistic effect when combined with bupivacaine (Devi et al. 2018). Adenosine has also been shown to play a key role in cartilage homeostasis (Mediero and Cronstein et al. 2013). Importantly, preclinical studies have shown the whole-body protective benefits of ALM solution cannot be achieved using each drug individually (Dobson and Letson 2016). It is envisaged that an ALM infusion or intra-articular bolus may benefit patients undergoing orthopaedic interventions, and other major surgery, by reducing surgical stress, preventing bleeding and promoting a more permissive environment that enhances tissue healing following major surgery (Dobson 2015, Davenport et al. 2017). The potential clinical application of ALM solution in orthopaedic surgery as a novel strategy for protecting native articular cartilage and improving patient outcomes is currently being investigated.

### Limitations

A potential limitation of the present study may be the use of chondrogenically differentiated human bone marrow derived MSC, rather than human primary chondrocytes or cartilage explants. However, Kalamegam et al. (2016) have shown chondrogenic differentiation of human bone marrow MSC using similar methods and culture supplements, with chondrocyte-like cells acquiring a polygonal morphology and increased expression of type II collagen within 7 days of culture and continued deposition of cartilaginous matrix over a 21-day period. We also acknowledge that different substrate surfaces were used for viability and cytokine assays (polystyrene) and histology of disc cultures (porous polycarbonate membrane), though chondrogenesis of MSC has been demonstrated for both (Kalamegam et al. 2016, Murdoch et al. 2009). Our findings suggest, even with direct contact for up to 4 h, exposure to ALM solution had no adverse effect on chondrocyte-like cells in culture. However, in vitro studies cannot represent the complexities of the joint microenvironment and it is possible that the phenotypic responses we found in response to ALM solution may differ in vivo. Future studies that include more extensive investigation of inflammatory and oxidative stress responses of mature articular cartilage exposed to ALM solution in vivo are warranted and are the focus of current research.

## Conclusions

We conclude that short-term exposure of human chondrocyte monolayers to ALM solution improved viability compared to saline alone. In addition, ALM did not stimulate secretion of key inflammatory mediators or matrix-degrading MMP that are believed to delay healing and promote secondary injury.
